# *In vitro* simulation of erosive challenges to human enamel using a novel artificial mouth

**DOI:** 10.1002/cre2.111

**Published:** 2018-06-19

**Authors:** Abubaker S. Qutieshat, Andrew Graham Mason, Richard Graham Chadwick

**Affiliations:** ^1^ Assistant Professor of Conservative Dentistry, Department of Conservative Dentistry Jordan University of Science & Technology Jordan; ^2^ Senior Clinical Lecturer, School of Dentistry University of Dundee UK; ^3^ Professor of Operative Dentistry and Applied Dental Material Science, Honorary Consultant in Restorative Dentistry, School of Dentistry University of Dundee UK

**Keywords:** dental, Erosion, evaluation, simulation

## Abstract

This *in vitro* work reports upon the design, build and operation of an artificial environment (Saltus) that sought to simulate the process of in vivo dental erosion upon human enamel. A novel testing environment, housed 8 erosion testing substrate specimens, that on separate occasions were subject to 4 different experimental diets, of increasing erosive challenge, simulating the consumption of an acidic beverage. Each set of specimens was subjected to one of the experimental diets only. These were liquid only and administered the test beverage over a standardized range of volumes and durations. Flow of both artificial unstimulated and stimulated saliva was maintained throughout and the effects upon the substrates were measured by profilometry, surface microhardness determination and chemical analysis of the saliva and beverage mixture for traces of Calcium and Phosphate ions. The overall trend of surface hardness reduction, depth of surface loss and ion loss across the diets increased in proportion to the severity of insult. Accepting the limitations of this study Saltus appeared to perform well as an environment in which to simulate and assess dental erosion using parameters defined by previous *in vivo* observations of human drinking behaviour. The authors however acknowledge that in vitro testing can never replicate fully the in vivo situation.

## 1 INTRODUCTION

A contemporary definition of dental erosion, based upon current perceptions, knowledge and concepts, defines erosive dental wear as a chemical mechanical phenomenon where loss of tooth structure occurs from both acidic attack and mechanical forces (Lussi & Carvalho, 2014)^.^ This differentiates it from dental caries where the acid responsible is produced by the fermentation of carbohydrates by microorganisms that reside in dental plaque attached to the surface of the tooth.

While a food or drink that has a pH of 7 is considered neutral and not able to bring about direct acidic damage to the teeth, many foods and beverages are in fact acidic. This is because acid content in food and drinks is important for flavor, taste perception, product stability and shelf life (Chadwick, 2006). When assessing the erosive potential of foods or drinks, the quantity, duration and frequency of intake are the most important factors to be considered (Chadwick, 2006). Whereas it would be desirable to assess the erosive potential of a food/beverage *in vivo* current validated methods are unable to do this readily. Theoretically speaking, such a procedure would require studies of long duration and would always have the challenge of controlling exposure of the tooth and preventing other factors from affecting the tooth's' surface, such as, for example, other tooth wear mechanisms such as abrasion and attrition (West, Davies, & Amaechi, 2011).

In any *in vitro* model, a guided standardization of experimental protocols can easily be made, which offers the potential to examine one variable at a time or, if desired, other variables can be introduced into the model. Furthermore, the ability to accurately measure an eroded tooth surface *in vitro* using precise devices and techniques favors such an approach compared to the *in vivo* setting.

In general, a lack of a standardization among different *in vitro* and *in situ* erosion testing experiments render them non comparable as a result of different experimental variables (West et al., 2011). In an endeavor to overcome this, parameters for a realistic erosion testing regime based upon natural human drinking behaviour have been developed derived from behavioral observations (Qutieshat, Mason, & Chadwick, 2015). Often *in vitro* erosion testing regimes use extracted human teeth as the substrate upon which the effects of dental erosion are observed. These often use unrealistically long exposure times to gain a measurable effect, that do not take account of normal drinking behaviour and effects of saliva (Cochrane, Cai, Yuan, & Reynolds, 2009; Jensdottir, Bardow, & Holbrook, 2005). In the past an artificial mouth has been used to assess the effect of mineral supplements to citric acid upon bovine enamel but the basis of its protocol of operation, that sought to mimic normal sipping behaviour, was not given (Attin, Meyer, Hellwig, Buchalla, & Lennon, 2003).

The purpose of this work was to report upon the design, build and operation of an environment that sought to simulate *in vitro* the process of *in vivo* dental erosion upon human dental enamel. It aimed to permit control of the variables of beverage consumption volume and flow rate as well as both the composition and flow rate of artificial saliva.

## 2 MATERIALS AND METHODS

### 2.1 Testing environment – design, build and testing

The testing apparatus, Saltus, was designed using CAD CAM drawings (Auto CAD 2012, Autodesk Inc., San Rafael, California, USA) translated into 3D virtual models using two software packages (Blender ™ 2.72, Stitching Blender Foundation, Amsterdam, The Netherlands and SketchUp 2013, Trimble Navigation Limited, Sunnyvale, CA, USA) that permitted modeling of projected fluid flow, component modification and finally manufacture from Perspex acrylic sheets precision cut under computer control by an ILS‐III NM Intelligent Laser Cutting system (Laser Tools and Technics Corp., His‐Chu City, Taiwan). The resultant components were then assembled using a watertight two‐component polymeric cement (Tensol 70, Perspex Distribution Ltd., Blackburn, UK).

**Figure 1:**
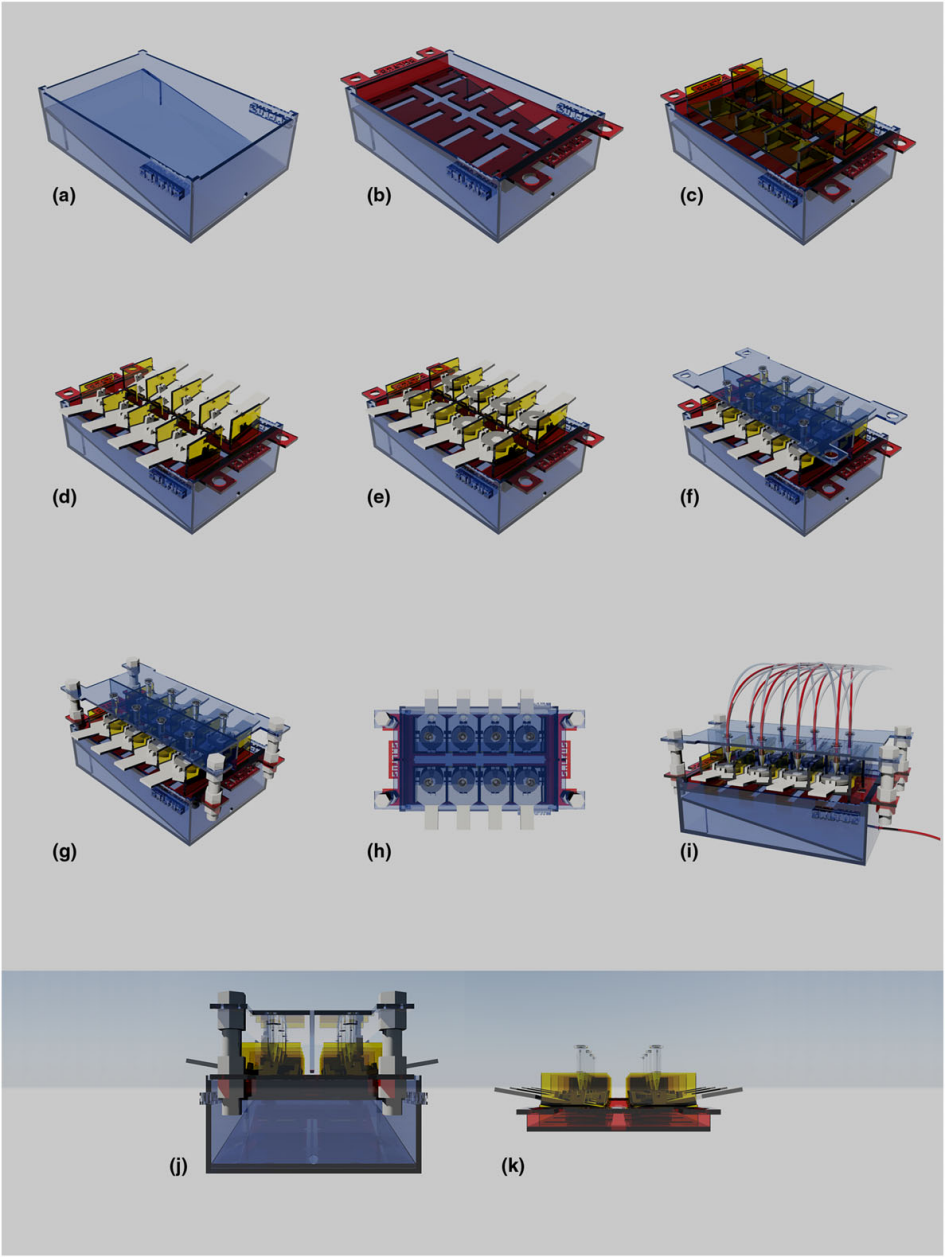
AutoCAD modeling and drafting of Saltus. (A) Body: A reservoir that consisted of a 10° sloping surface, drainage outlet and tray‐holding slots. (B) Tray: A base, onto which shelves sat, consisting of drainage holes, handles and shelf‐holding slots. (C) Two shelves: Each shelf consisted of 4 cells. The shelf base is sloping towards the centre by 10°. (D) 8 Specimen disk holders: Each consisted of a handle, an anti‐slope step and sample‐holding slots. Each holder was designed to receive 1 specimen disk (E) of 3 mm in thickness and 30 mm in diameter. (F) Roof: This formed the tubing/model interface and consisted of tubing inlets, sample‐set separator and adjustable height pedicles (G). (H) Top view. (I) Source and clearance tubing. (J) Side view. (K) Mixer/specimen configuration

The final apparatus (Figure 1), consisted of;

- A body – a fluid reservoir comprising an enclosed 10° sloping surface, drainage outlet and tray holding slots
- a tray – a base onto which the sample holders sat; comprising drainage holes, handles and shelf‐holding slots
- a roof – a tubing/model interface with tubing inlets, sample‐set separators and adjustable height pedicles
- two shelves – each housing 4 experimental cells. Each sloped downwards towards the centre of the apparatus by 10° to foster fluid flow
- Eight specimen holders – each capable of holding a prepared erosion testing substrate disc 3 mm thick and 30 mm in diameter.

To this was affixed a fluid circulating system capable of delivering, in a controlled manner, test beverages and both artificial unstimulated and stimulated saliva recipes. The principal pump used was a 24 channel drive peristaltic pump (Isamatec IPC 24, Michael Smith Engineers Ltd., Wetherby, West Yorkshire, UK) that propelled the fluids through a system of peristaltic (Michael Smith Engineers Ltd., Wetherby, West Yorkshire, UK) and stainless steel hypodermic (Shannon Coiled Springs Ltd., Limerick, Republic of Ireland) tubing mixing them at the point of exit above the test specimen. Table 1, based upon previous published work ^(4, 8–10)^, summarizes the guide flow rates to be achieved by Saltus. For practical convenience (pump speed and available tubing diameters) this *in vivo* value was rounded to 15 ml min^−1^.

**Table 1:** Summary of Flow Rates to be achieved in Saltus

FLUID VARIABLE	FLOW RATE (ml/min)
Drinking rate (Qutieshat et al., 2015) with	13.3
Sip volume = 16.8 ml	(Approximated to observed behaviour (Qutieshat et al., 2015) for uniform flow)
Temperature = 14.9°C	
For full dose diets – total daily volume = 660 ml, Administration time of beverage = 44 minutes.	
For half dose diets – total daily volume = 330 ml, Administration time of beverage = 22 minutes.	
Stimulated saliva flow rate (Dawes, 1970; Dawes, 1987)	5.0
Unstimulated saliva flow rate in waking hours (Thomson, Chalmers, Spencer, & Slade, 2000)	0.3
Unstimulated saliva flow rate in sleeping hours (Thomson et al., 2000)	0.1

Such delivery was achieved by a combination of correct selection of tubing diameter for permissible flow rate range and speed (Revolutions per minute (RPM)) of the peristaltic pump for:

$$\mathrm{RPM}={\text{Expected Flow Rate}}^{*}\;\left(\text{maximum pump drive speed}/\text{maximum tube flow rate}\right)$$

All tubing terminated in a mixing tip, located immediately above and perpendicular to, each specimen holder, formed from modified Eppendorf tubes (Cole‐Parmer Instrument Co. Ltd., Hanwell, London, UK) with an exit port 2.79 mm in diameter. These were bonded to the roof section of Saltus using Sheramega 2000 adhesive (Shera Werkstoff – Technologie Gmbh & Co., KG, Lenforde, Germany).

Prior to delivery to Saltus the beverages and saliva's were housed in separate reservoirs;

*Beverage Reservoir*– Prior to reaching the peristaltic pump for distribution the test beverage was stored in a 5 liter capacity polyethylene aspirator with tap (Azlon, Scilabware Ltd., Stoke‐on‐Trent, Staffordshire, UK) in a mini fridge (Thermoelectric Cooler and Warmer, Diplomat, Slemcka Ltd., Smethwick, Birmingham, UK) operating at 14°C. A submersible low voltage direct current fountain pump (Vovyo Technology Co., Ltd., Shenzhen, Guangdong, China), with a flow rate of 2.65 liters min^−1^ controlled movement of the beverage through Tygon pump tubing (Cole‐Parmer Instrument Co. Ltd., Hanwell, London, UK) from this to a 5000 ml glass beaker (Fisher Scientific UK Ltd., Loughborough, UK) from which it was conveyed onwards by the peristaltic pump. The timing of the fountain pump, by a micrometer timer switch (ZYT16G, Shanghai Zhuoyi Electronic Co. Ltd., Pudong, Shanghai, China), was dependent upon the drinking behaviour being simulated. Such an arrangement was necessary to minimize degassing of carbonated beverages. Their fizzy nature necessitated the submersion of the Tygon pump tubing in the beverage reservoir using a 60 g fishing weight (WSB Tackle Ltd., Redruth, Cornwall, UK) so as to prevent air entrapment within it.
  Based upon a previous observational study (Qutieshat et al., 2015) the intended temperature of the drink at delivery to the erosion testing substrate was to be around 14.9°C.
*Saliva reservoirs*– The peristaltic pump delivered either unstimulated or stimulated artificial saliva to the erosion test substrates at appropriate times and flow rate. Table 2 gives the origin and composition of these (Leung & Darvell, 1991; Leung & Darvell, 1997). Each was housed in separate 2000 ml laboratory glass bottles (Fisher Scientific UK Ltd., Loughborough, UK) that were sealed to prevent the escape of carbon dioxide which, as well as affecting the chemical stability of the solution, would also deplete its carbonate content. Flow of the solutions from the reservoir to the peristaltic pump was through stainless steel hypodermic tubing (Shannon Coiled Springs Ltd., Limerick, Republic of Ireland) ‐ 22‐R gauge for unstimulated and 14‐T gauge for stimulated.

**Table 2:** The Composition of Saliva's used

	Concentration	
*Stimulated saliva Recipe*	g/L	Mol/L
**Stock solution A**		​
NaH_2_PO_4_ (Sigma, MO)	32.13	0.233
KCl (Sigma, MO)	86.0	1.164
NaCl (Sigma, MO)	7.21	0.123
NH_4_Cl (Fisher Scientific, UK)	11.0	0.205
Trisodium citrate di‐hydrate (Sigma, MO)	1.1	3.74 × 10^−3^
Lactic acid (Acros organics)	2.9 ml	0.039
**Stock solution B**		​
Urea (Sigma, MO)	5.0	0.167
Uric acid (Acros organics)	0.375	4.46 × 10^−3^
NaOH (Sigma, MO)	0.1	5.00 × 10^−3^
**Stock solution C**		​
KSCN (Fisher Scientific, UK)	12.0	0.123
**Working Solution**		​
CaCl_2_.2H_2_O (Sigma‐Aldrich, MO)	2.4 g/L	​
**Final pH 7.15**		

	Concentration	
*Stimulated saliva Recipe*	g/L	Mol/L
**Stock solution A**		​
NaH_2_PO_4_ (Sigma, MO)	32.13	0.233
KCl (Sigma, MO)	86.0	1.164
NaCl (Sigma, MO)	7.21	0.123
NH_4_Cl (Fisher Scientific, UK)	11.0	0.205
Trisodium citrate di‐hydrate (Sigma, MO)	1.1	3.74 × 10^−3^
Lactic acid (Acros organics)	2.9 ml	0.039
**Stock solution B**		​
Urea (Sigma, MO)	5.0	0.167
Uric acid (Acros organics)	0.375	4.46 × 10^−3^
NaOH (Sigma, MO)	0.1	5.00 × 10^−3^
**Stock solution C**		​
KSCN (Fisher Scientific, UK)	12.0	0.123
**Working Solution**		​
CaCl_2_.2H_2_O (Sigma‐Aldrich, MO)	2.2 g/L	​
**Final pH ​6.85**		

#### 2.1.1 Assessment of Mixers

Prior to conducting any erosion research, and in order to establish that the mixers of Saltus were effective, two different chemical solutions were prepared – 100 ml of a 1 M Citric Acid (Sigma Aldrich Ltd., Gillingham, Dorset, UK) Solution and 0.005 g Bromophenol Blue (Sigma Aldrich Ltd., Gillingham, Dorset, UK) in 500 ml of deionized water. These were thoroughly mixed together in the laboratory using a magnetic stirrer (Stuart SM1, Keison Products, Chelmsford, Essex, UK) and a collected 1000 μl sample was passed through a spectrophotometer (WPA Lightwave, $2000 U*V*/Vis, Biochrom Ltd., Cambridge, UK) and its absorption value, at ƛ = 590 nm, set to read 0.000. The same unmixed solutions were passed simultaneously through Saltus and samples of the resultant mixture were collected from the exit ports of the mixers. Spectrophotometry was repeated. Bromophenol blue dye is an acid base indicator and the citric acid solution is clear in color. At a low pH, the dye absorbs both ultraviolet and blue light most strongly and so appears yellow in a well‐mixed solution. The hypothesis tested by this method was that mixing Bromophenol blue with citric acid would yield a yellow solution if mixing was efficient. This experiment was repeated four times.

*Specimen preparation of human enamel* ‐ caries free extracted human permanent molars, collected under the governance of the Tayside Biorepository prior to September 2006, were selected from the dental schools research tooth collection. These had been removed as part of planned clinical care and donated for research. They were stored in distilled water following disinfection in sodium hypochlorite. The roots of each tooth were removed just above the cemento‐enamel junction and the remaining coronal portion of the tooth was embedded in acrylic resin aligning it vertically in cylindrical molds 3 cm in diameter. Sagittal slices, 1 mm thick, were cut in a mesial – distal direction using a slow speed diamond saw (Isomet Buehler Ltd., USA), at 450 RPM, under water coolant until the first signs of enamel were observed in the sections. Thereafter 3 mm thick slices were prepared. Only samples containing no visible dentine were selected for use in this work following lapping the specimen flat using a PM5 precision lapping and polishing machine (Longitech, Glasgow, Scotland) and a slurry of calcined aluminium oxide powder with a particle size of 9 μm (Longitech, Glasgow, Scotland). Such samples were placed on the sample holders of Saltus. A typical sample of enamel acquired in this manner measured 7 × 7 mm at its exposed surface.

#### 2.1.2 Characterization of enamel specimens

Prior to and following exposure to an erosive beverage the surface hardness and surface profile of each specimen were determined.

*Surface micro hardness* – A TIV (Through Indenter Viewing) hardness tester (GE Measurement & Control, Groby, UK) with Vickers diamond, under a 9.8 N load, was used to measure the surface hardness at 10 sites per specimen. From these the percentage relative hardness of each specimen was calculated using the formula.

$$\%\text{Relative Hardness}={\left(\text{Mean Final Hardness}/\text{Mean Initial Hardness}\right)}^{*}\;100$$

The site of each indent was confirmed by visualization through the TIV not to be in close proximity to other measurements taken of the sample.

*Surface Profile*– The specimens were profiled using a surface contacting profilometer (Planer SF220. Surface Profiler, Planer Products Ltd., Sunbury on Thames, UK) with a diamond stylus, of 20 μm tip diameter, moving along a straight line at 10 mm min^−1^ across the mid‐point of the specimen. The surrounding flat specimen mounting epoxy served as a datum against which the erosion surface depth loss was measured. For each state each sample was profiled three times and a mean depth loss calculated relative to the surrounding mounting epoxy resin by the instruments software.

The erosive effects of each diet upon the specimens, in addition to determination of surface profile and microhardness, were assessed by performing chemical analysis of the saliva beverage mixture solution, collected in a reservoir (10 L tap‐equipped Polyethylene aspirator), for traces of calcium and phosphate ions using an automated chemistry analyzer (ADVIA® 2400 Clinical Chemistry System, Siemens Healthcare, Cumberly, UK) from three 10 ml collections of this fluid collected prior to and following the introduction of the erosion testing samples into Saltus. Following collection these were stored at 4°C in 10 ml universal vials. The mean values for each ion were used to calculate by subtraction the concentrations of eluted ions arising from exposure to the erosive beverage.

#### 2.1.3 Erosion testing regime

In this work a range of drinking behaviors, that exposed the erosion test substrate to an erosive challenge, were simulated and their effects upon human enamel were investigated. These looked at both different daily volumes of consumption and their duration. The erosion cycles, termed diets, used were informed by a previously published observational study (Qutieshat et al., 2015) and were delivered to the specimens using Saltus. For the purposes of this work a diet is a program delivered by Saltus that is comprised of a series of cycles through which erosion substrates were exposed to artificial saliva and a test beverage. Diets were of the duration of 5 or 7 days. For each diet the experiment was repeated twice upon new specimens. Operation of Saltus was continuous over each 24 hour period of operation and consisted of 3 daily periods;

- A day “waking hours” period
- A night “sleeping hours” period
- A stimulated period.

During the day and night periods artificial unstimulated saliva circulated through the system at physiological flow rates, while during the stimulated period artificial stimulated saliva flowed at higher rates simultaneously with the test beverage (Coca‐Cola Regular, Coca‐Cola Enterprises Ltd., Uxbridge, UK, pH = 2.47 _±_ 0.02). Table 1 summarizes the test beverage kinematic behaviour values, consumption quantity, drinking time period and beverage temperature and also the flow rates of the artificial salivas. A total of 4 different diets were evaluated in this work.

*Diet 1: Short‐Single dose diet*: this diet was executed over a period of 5 days, the 1st and the last days were rest cycles while the 2nd, 3rd and 4th days were test cycles. Test cycles consisted of 1 can (330 ml) of the test beverage administered over a duration of 22 min. Thus in all, 3 cans were consumed per sample in this diet.
*Diet 2: Long‐Single dose diet:* this diet was executed over a period of 7 days, the 1st and the last days were rest cycles while the 2nd, 3rd, 4th, 5th and 6th days were test cycles. Test cycles consisted of 1 can (330 ml) of test beverage administered over the duration of 22 min a day. Thus in all, 5 cans were consumed per sample in this diet.
*Diet 3: Short‐Double dose diet*: this diet was executed over a period of 5 days, the 1st and the last days were rest cycles while the 2nd, 3rd and 4th days were test cycles. Test cycles consisted of 2 cans (660 ml) of the test beverage administered over a duration of 44 min. Thus in all, 6 cans were consumed per sample in this diet.
*Diet 4: Long‐Double dose diet*: this diet was executed over a period of 7 days, the 1st and the last days were rest cycles while the 2nd, 3rd, 4th, 5th and 6th days were test cycles. Test cycles consisted of 2 cans (660 ml) of test beverage administered over a duration of 44 min a day. Thus in all, 10 cans were consumed per sample in this diet.

It should be noted that for all of these diets a rest cycle comprised a 24 hour period during which unstimulated saliva flowed at a rate of 0.3 ml min^−1^, between the hours of 0700 to 2300 hours (Waking Hours), and from 2300 until 0700 hours (Sleeping Hours) at 0.1 mm min^−1^. These primarily served to facilitate ion collection rather than imitate human behaviour. On days when a test beverage was introduced into the system (Test Cycle) this pattern continued with the exception of between the hours of 1400–1500 during which stimulated saliva flowed at 5.0 ml min^−1^ and the test beverage was introduced according to the diet being administered.

To investigate any effects of diets (diet 1, 2, 3 and 4) and experimental run (Run 1 and 2) on the surface hardness change a 2‐way analysis of variance on this data was undertaken. A follow‐up analysis (Bonferroni post‐test) was undertaken to determine the effect of the diet factor over all the groups (diet 1, 2, 3 and 4).

To investigate any effects of diets (diet 1, 2, 3 and 4) and experimental run on the amount of surface loss a 2‐way analysis of variance on this data was undertaken. A follow‐up analysis (Bonferroni post‐test) was undertaken to determine the effect of the diet factor over all the groups (diet 1, 2, 3 and 4).

To investigate any effects of diets (diet 1, 2, 3 and 4) and ion type upon ion loss a 2‐way analysis of variance on this data was undertaken. A follow‐up analysis (Bonferroni post‐test) was undertaken to determine the effect of the diet factor over all the groups (diet 1, 2, 3 and 4).

For the purposes of comparison the observed depth of surface loss was divided by the duration of experimental run.

## 3 RESULTS

Prior to the introduction of any erosion substrates into the device analysis of the Calcium and Phosphate ion concentrations of the collected saliva mixture following a substrate and beverage free one day diet confirmed no effect upon the ionic composition of the saliva.

The % absorption spectrophotometer readings for bromophenol dye mixed with 1 M citric acid, using either the magnetic stirrer or Saltus, did not differ significantly (*P* > 0.05). This, together with the observation that the solution mixed in Saltus was yellow in color with no blue spotting, confirmed the efficiency of the devices mixing system.

**Figure 2:**
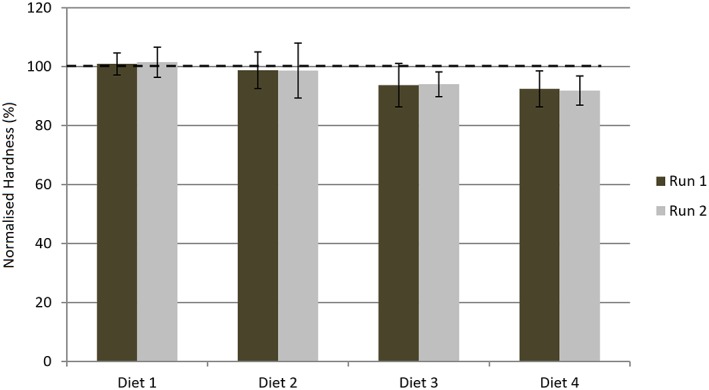
The mean post diet hardness values and their associated standard deviations, for human enamel samples, for each of the 4 diets administered on two experimental runs as expressed as a % of the pre‐diet hardness values. Note 100% represents no change in hardness value

Figure 2 summarizes the observed changes in surface hardness following the 4 diets upon the human enamel samples administered on two experimental runs. The mean Vickers hardness values for all the test substrates prior to exposure to the erosive beverage were 278.5 (S.D. = 36.8). It is clear that a dose response relationship exists between the quantity of consumption and the observed mean percentage reduction in surface hardness.

**Figure 3:**
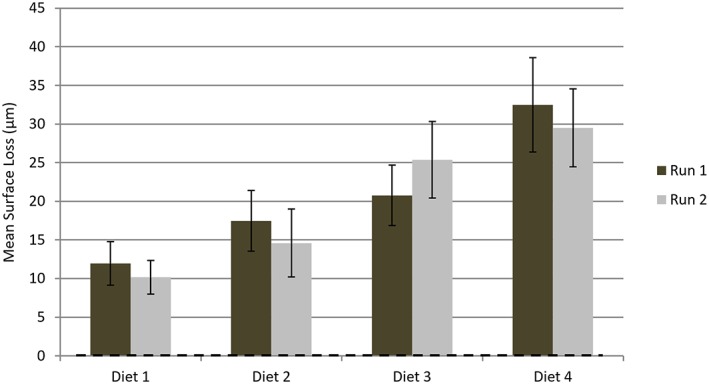
The mean surface loss (μm) and their associated standard deviations, relative to pre‐diet values, for human enamel samples, for each of the 4 diets administered on two separate experimental runs. Note: a positive loss value represents a loss of material

Figure 3 summarizes the mean surface loss (μm) and their associated standard deviations, relative to pre‐diet values, for human enamel samples, for each of the 4 diets administered on two separate experimental runs. Table 3 presents this data as a mean value of depth of surface loss per hour for each diet. It is clear that a dose response relationship exists between the quantity of consumption and the rate of surface loss.

**Table 3:** The mean observed depth of surface loss (μm) per hour (derived for comparative purposes by dividing the post diet surface loss by the diets duration), with associated standard deviations, for each diet and erosion substrate.

SUBSTRATE	Diet 1	Diet 2	Diet 3	Diet 4
Human Enamel	10.06 (1.15)	8.75 (1.11)	20.98 (2.96)	16.90 (2.09)

**Figure 4:**
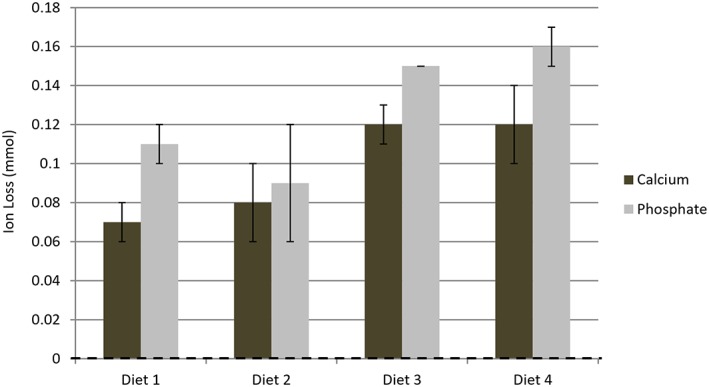
The overall mean ion loss (mmol/day) and their associated standard deviations for Calcium and Phosphate following the 4 diets

Figure 4 gives the overall mean ion loss (mmol/day) and their associated standard deviations for Calcium and Phosphate following the 4 diets.

For each measured parameter separate two way analyses of variance were undertaken to determine if the different diets, experimental runs and their interaction had any statistically significant effects. In all cases no effect overall of experimental run or interaction of diet and experimental run was found (*P* > 0.05). The effect of the different diets was however highly statistically significant (*P* < 0.001) for all parameters. Bonferroni post testing revealed that:

- The % surface hardness reductions differed significantly (*P* < 0.05) for diet 1 relative to diets 3 and 4 on both experimental runs. No other significant differences were found. There was thus generally good agreement between the experimental runs.
- The observed differences in surface loss was only statistically significant (*P* < 0.01) in the cases of Diet 1 *versus* Diets 3 & 4, Diet 2 *versus* Diet 3, Diet 3 *versus* Diet 4 following run 1. In the case of run 2 this was also the case with the exception of Diet 3 *versus* Diet 4 where there was no statistically significant difference. Reproducibility between the runs was therefore good.
- For all ions (Calcium and Phosphate), with the exceptions of Diet 1 *versus* Diet 2 and Diet 3 *versus* Diet 4 significant differences (P < 0.05) were seen between the diets.

## 4 DISCUSSION

The primary aim of this study was to design, build and evaluate a novel artificial mouth for use in the evaluation of dental erosion *in vitro* based upon behavioral observations. It was therefore not the aim at this stage to use it to evaluate a range of drinking habits more to illustrate the models utility. Before considering the findings revealed by the pre and post exposure measurements of the erosion substrate it is important to appreciate the performance of Saltus and the measurement techniques employed in the study. In terms of Saltus function it is reassuring to note that no changes in the assessed ionic composition of the circulating fluids occurred when no testing substrates were present in the apparatus. In addition, the experiment to assess efficiency of mixing of fluids confirmed mixer efficiency. The technique used to assess this may prove useful to other researchers in diverse fields within and out with dentistry. It should be noted that proteins and other inorganic substances were left out of the formulation due to being difficult to characterize and obtain reproducibly though it is acknowledged that *in vivo* these proteins are important. It is also important to note that in the present model the flow rate, pH and chemical composition of the stimulated and unstimulated saliva differed. This work used a contacting surface profilometer to assess depth of erosive loss. Clearly if incorrectly applied this has the potential to scratch the demineralised substrate surface giving an erroneous result. Inspection under magnification however of the profiled specimens demonstrated that this was not the case. To the authors knowledge this was the first time an automated through indenter viewing (TIV) hardness tester had been applied in dental research. Its application was far less fatiguing than the researchers past experience, over many years, of using a conventional Vickers hardness microscope. It thus afforded many more measurement possibilities together with magnification and, in fact, this project would not have been feasible in its extent were such an instrument unavailable. It's software settings covered a possible measurement range of 30–500 Vickers hardness units.

The work sought to reproduce observed behaviour (Qutieshat et al., 2015) and inevitably due to engineering constraints (matching pump speed and tubing diameters) the observed physiological values for beverage flow rate were adjusted to a deliverable value. This may be of interest to other researchers planning such devices.

Although the present work was run in a highly controlled fashion it can be argued that the extent of the erosive lesion may be affected by inter‐structural variations of the specimens. This could arise for example by the presence of biological variation (e.g. different specimen sources, location and environmental history). In this study however, this is unlikely to be a factor for variability among specimens in terms of their hardness values standard deviations did not exceed 13.2% (Table 3). It has been previously reported in the literature that hardness deviations of up to 16% are to be expected for human enamel specimens (Turrsi, Messias, Corona, & Serra, 2010).

When surface loss is taken into account (Figure 3 and Table 3) the single‐dosed diets (Diet 1 and 2) had a surface loss rate of 8.75–10.06 μm/hr for human enamel. This corresponds to almost half the surface loss rate observed in double‐dosed diets (Diet 3 and 4) (16.90–20.98 and 12.08–14.91 μm/hr respectively) (Table 3). Clearly, a dose–response relationship between the quantity of consumption and the amount of surface loss was found. Figure 3 reflects this result but it is important to note that this looks at the total surface loss for each diet whereas Table 3 refers to the rate of loss per hour and therefore better reflects the dose dependent relationship. Not recognizing this point risks concluding erroneously that the amount consumed has a higher impact than the duration of consumption. A similar dose–response relationship was reported recently in a study that assessed the association between dental erosion and several dietary risk indicators such as the amount of consumption (Sovik, Skudytyte‐Rystaad, Sandvik, & Mulic, 2015). Although the total amount of loss increased from short‐ to long‐diets (i.e. from Diet 1 to 2 and from Diet 3 to 4); the hourly surface loss rates were comparable as was expected in view of the standardized test‐cycles that the artificial mouth system was programmed to deliver.

In light of the above there is a case for a combined hardness and profilometric evaluation of the results for all the post‐diet relative hardness percentage values observed for human enamel were greater than 90% despite the measured surface loss. If erosion assessment relied solely upon hardness testing it might lead to false conclusions ‐ such as assuming that the test beverage had no effect upon human enamel especially in the cases of Diets 1 and 2. Clearly, this was not the case for surface loss was in fact detected (in the range 10.18 ± 2.17 to 17.48 ± 3.93 μm) for diets 1 and 2. It is possible that during the test cycle the test substrate underwent surface softening, due to erosion, and the resultant softened surface was lost prior to the measurement of surface hardness of what essentially would be a relatively harder and un‐eroded fresh surface. Such an entity has been observed by others (Van Eygen, Vannet, & Wehrbein, 2005).

In addition to assessing the surface characteristics of the teeth following exposure to a regular cola beverage the present work also looked at the elution of calcium and phosphorous ions from them. Other *in vitro* erosion testing models have also done so in relation to human enamel (Cochrane et al., 2009; Jensdottir et al., 2005; Larsen & Richards, 2002; Willershausen & Schulz‐Dobrick, 2004). Two of these (Cochrane et al., 2009; Jensdottir et al., 2005) adopted a prolonged exposure‐by‐immersion time period of 24 hours which, although registering an effect, was not founded upon normal physiology and drinking behaviour. Another study reported that calcium ion loss per hour of acidic exposure was 0.46 mmol/l (Larsen & Richards, 2002) and its findings were in accord with another study (Xavier, Rai, Hegde, & Shetty, 2015) that utilized demineralisation‐remineralisation cycling and also demonstrated calcium ion loss of 0.43 mmol/l per hour. The same study also reported phosphate ion loss to be 0.52 mmol/l per hour. In the present work, human enamel calcium loss in Diets 1 and 2 was in the range of 18.2–20.7 mmol/l and the phosphate loss was in the range of 25.6–30.9 mmol/l. This is in line with the conclusion of an *in vitro* study that assessed human enamel mineral loss upon the exposure to a regular cola beverage. Here the dental substrates were found to lose a stable ratio of calcium and phosphate throughout the erosive process (Willershausen & Schulz‐Dobrick, 2004). In the present work a dose–response relationship was observed where single‐dosed diets lost significantly less ions relative to double‐dosed diets.

Accepting the limitations of this study Saltus performed well as an environment in which to simulate and assess pure dental erosion though it is acknowledged that a contemporary definition of erosive tooth wear (Lussi & Carvalho, 2014) describes it as a chemical mechanical phenomenon and the latter is absent in the model. Unlike a previously reported artificial mouth (Attin et al., 2003) used to assess the effect of mineral supplements to citric acid upon bovine enamel erosion, it permitted the simultaneous flow of different types of artificial saliva (stimulated and unstimulated) and erosive beverage and as such, together with the evidence based drinking behaviour that informed its operation (Qutieshat et al., 2015), offers an improvement in such laboratory simulation. Notwithstanding this it is hoped that the techniques described in its design will be of value to other researchers. The authors however acknowledge that in vitro testing can never fully replicate the *in vivo* situation but this apparatus offers an improvement in such simulation.
